# Potential association between obstructive lung diseases and cognitive decline

**DOI:** 10.3389/fimmu.2024.1363373

**Published:** 2024-07-22

**Authors:** Magdalena Figat, Aleksandra Wiśniewska, Jacek Plichta, Joanna Miłkowska-Dymanowska, Sebastian Majewski, Michał S. Karbownik, Piotr Kuna, Michał G. Panek

**Affiliations:** ^1^ Department of Internal Medicine, Asthma and Allergy, II^nd^Chair of Internal Medicine, Medical University of Lodz, Lodz, Poland; ^2^ Department of Clinical Pharmacology, I^st^Chair of Internal Medicine, Medical University of Lodz, Lodz, Poland; ^3^ Department of Pneumology, Medical University of Lodz, Lodz, Poland; ^4^ Department of Pharmacology and Toxicology, Medical University of Lodz, Lodz, Poland

**Keywords:** neurodegeneration, asthma, COPD, cognition, PKA, CREB, inflammation, brainlungs crosstalk

## Abstract

**Introduction:**

Chronic obstructive lung diseases, such as asthma and COPD, appear to have a more extensive impact on overall functioning than previously believed. The latest data from clinical trials suggests a potential link between cognitive deterioration and chronic obstructive inflammatory lung disease. This raises the question of whether these diseases affect cognitive functions and whether any relevant biomarker may be identified.

**Methods:**

This prospective observational study included 78 patients divided equally into asthma, COPD, and control groups (n=26, 27 and 25 respectively). The participants underwent identical examinations at the beginning of the study and after at least 12 months. The test battery comprised 16 questionnaires (11 self-rated, 5 observer-rated, assessing cognition and mental state), spirometry, and blood samples taken for PKA and CREB mRNA evaluation.

**Results:**

A 2.3-fold increase in CREB mRNA was observed between examinations (p=0.014) for all participants; no distinctions were observed between the asthma, COPD, and control groups. Pooled, adjusted data revealed a borderline interaction between diagnosis and CREB expression in predicting MMSE (p=0.055) in COPD, CREB expression is also associated with MMSE (β=0.273, p=0.034) like with the other conducted tests (β=0.327, p=0.024) from COPD patients. No correlations were generally found for PKA, although one significant negative correlation was found between the first and second time points in the COPD group (β=-0.4157, p=0.049),.

**Discussion:**

Chronic obstructive lung diseases, such as asthma and COPD, may have some linkage to impairment of cognitive functions. However, the noted rise in CREB mRNA expression might suggest a potential avenue for assessing possible changes in cognition, especially in COPD; such findings may reveal additional transcription factors linked to cognitive decline.

## 1 Introduction

Both asthma and chronic obstructive pulmonary disease (COPD) are chronic inflammatory lung diseases characterized most notably by obturation, inflammation, chronicity and heterogeneity. These are fundamental features in both conditions.

In asthma, upon exposure to allergens or during hyperventilation, immunoglobulin E (IgE) triggers the degranulation of mast cells (1) and enhances the secretion of Interleukin-6 (IL-6) (2). This IL-6 regulates and activates CD4+ T-helper 2 (Th2) lymphocytes, prompting them to produce *Th2 cytokines (* 2–4): IL-4, IL-5 and IL-13. Production of Th2 cytokines might be regulated by epithelial alarmins, for instance IL-25 and IL-33, which could have crucial role in non-atopic asthma (1). One of these cytokines, IL-5, stimulates the secretion of eosinophils (1), which are directed to the airways by eotaxin and the RANTES chemokine (Regulated upon Activation, Normal T cell Expressed and Secreted), also known as CCL5 (5). The inflammation in asthma has a chronic nature; this may be due to the activity of the NF-κB transcription factor, which amplifies and prolongs the inflammatory response (6). This results in increased local secretion of eosinophil-specific chemokines and reduced production of anti-inflammatory interleukin-10 (IL-10) (1, 6).

In COPD, exposure to cigarette or biomass smoke activates NF-κB and p38-mitogen activated protein kinase (MAPK) (7), increasing the presence of neutrophils and macrophages in the airways (1, 7). These cells release numerous mediators, including elevated levels of IL-8 (8), IL-1β, IL-6, tumor necrosis factor-α (TNF-α), and elastic enzymes (1, 8). Neutrophils promote neutrophilic inflammation and serine protease activity (1), both of which are implicated in common complications of COPD such as emphysema (1, 9) and mucus hypersecretion (5).

However, another important mediator of inflammation in chronic inflammatory lung diseases has been less widely investigated. In untreated or steroid-dependent asthmatic patients, inflammation severity has been found to correlate with increased expression of cAMP response element binding protein (CREB) (10). Similarly, CREB expression may also be elevated in COPD patients who respond poorly to inhaled corticosteroid (ICS) therapy (11). Furthermore, CREB activation appears to enhance airway smooth muscle (ASM) proliferation (12, 13).

**Figure 1:**
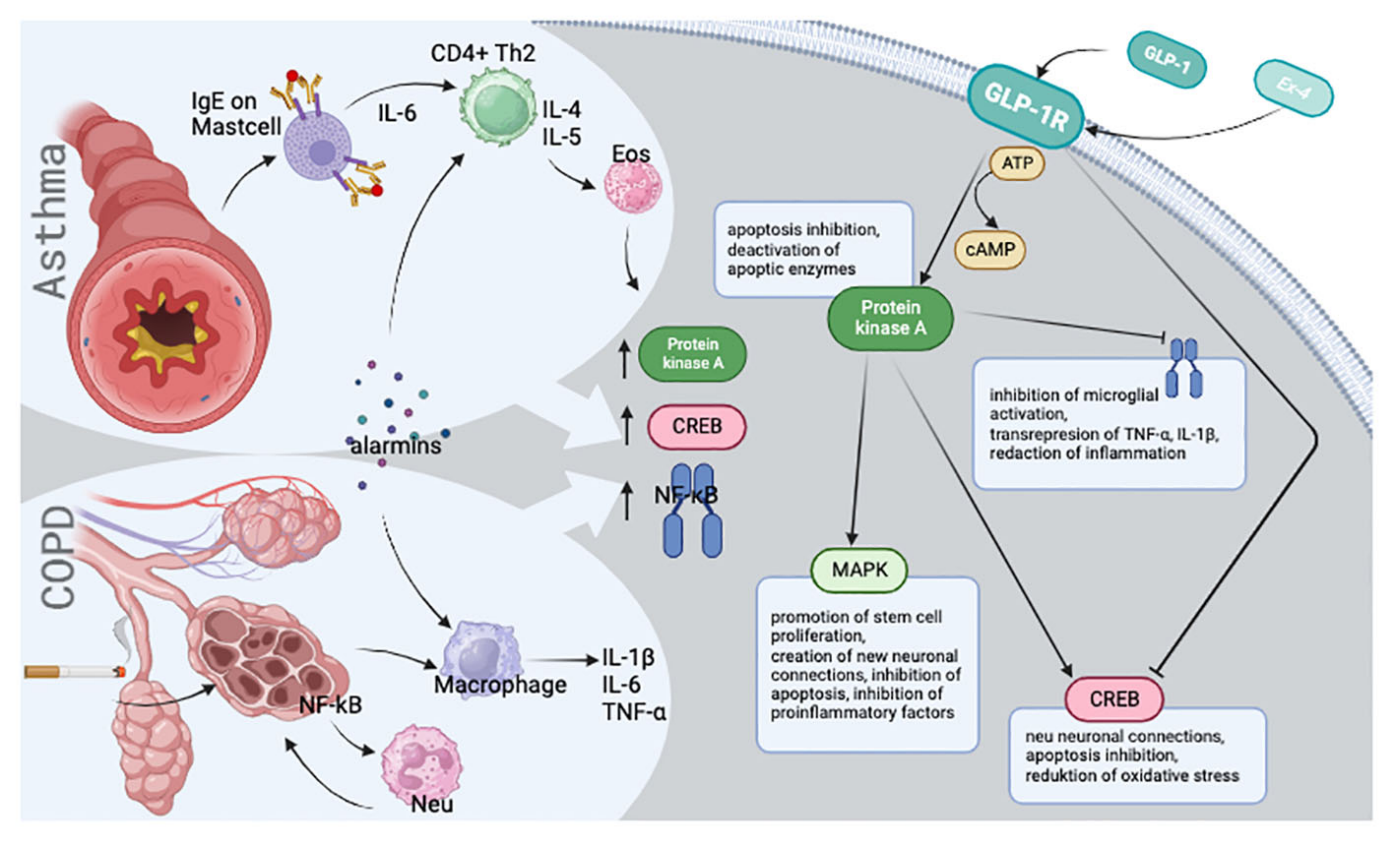
Visual presentation of the simplified disease pathomechanism and molecular background.

Both NF-κB and CREB are integral to the cAMP/PKA/CREB signaling pathway (6) (**Figure 1**). Upon stimulation of G protein-coupled receptors, such as glucagon-like peptide-1 receptor (GLP-1R) (14) or β2-adrenoreceptor (6), CREB level is decreased as cAMP levels increase (15). Notably, after stimulating GLP-1R with the exogenous analogue exendin-4 (16), NF-κB levels decreased (17), suggesting that elevated protein kinase A (PKA) expression may result in NF-κB depletion (17). Collectively, glucagon-like peptide-1 (GLP-1) appears to mitigate chronic lung inflammation by inactivating PKA-dependent NF-κB (18), reducing IL-8 mRNA expression in ASM cells (19). PKA stimulation may also influence the ability of asthma medications to inhibit abnormal ASM growth (20). Currently, no studies specify typical PKA expression levels in asthma or COPD.

GLP-1 moderates eosinophil activation (21) and mitigates bronchial hyperresponsiveness post-sensitization in asthma (21). Notably, GLP-1R expression is markedly diminished in the ASM of COPD patients, yet its overexpression significantly inhibits the release of inflammatory mediators (22, 23). While GLP-1 is known to reduce inflammation in both cases, it is not known whether GLP-1 levels rise or fall during chronic inflammatory diseases; this may be due to the poor availability of tissue suitable for study, as GLP-1 is be expressed in peripheral blood, but in the pancreas tissue, which requires biopsy.

According to the *outside-in* theory (24, 25), inflammation may not be confined solely to the lungs but could manifest throughout the body. This theory proposes that IL-6 may play a role in brain tissue reactivity among dementia patients (26) by promoting neuroinflammation. IL-1β is believed to amplify p38-MAPK activity, potentially resulting in Tau-hyperphosphorylation (26). Additionally, the TNF-α released from microglia (27), in conjunction with IL-6, has been linked to late-onset dementia and a greater risk of dementia (28, 29).

Several prospective studies have found a potential link between chronic inflammatory lung diseases and neurodegeneration (30–32). This relationship has also been identified in diffusion-weighted magnetic resonance imaging (30). However, although the molecular background remains unknown, the pulmonary conditions may double the risk of mild cognitive impairment or dementia as compared to the general population (33). Both threats predominantly emerge during immaturity (34) or during midlife (33). Prior research primarily sought epidemiological correlations without pinpointing the molecular basis, while recent approaches have focused on assessing cognitive changes in response to asthma medication adjustments (34, 35) or lung disease progression following anti-dementia treatments (36), with little interest in molecular mechanisms. Further research could enhance our understanding of the pathophysiology of inflammation in both scenarios, and open the door to more effective treatment strategies for each phenotype (chronic inflammatory lung diseases and neurodegenerative diseases). However, to better understand the molecular background, the first step is to evaluate the linkage between chronic obstructive lung diseases and cognitive deterioration.

The aim of the study.

The objective of this study was to:

1. Characterize the participants in each group with regard to epidemiological self-reported data, quality of life and disease; these are assessed by survey.
2. Determine cognitive functions using psychiatric assessment tests, and identify any relationships between cognition and the studied chronic inflammatory lung diseases.
3. Identify any relationships between cognitive evaluation and disease-specific questionnaires or spirometric parameters.
4. Examine the correlation between the PKA mRNA and CREB mRNA expression and the cognition assessment.

## 2 Materials and methods

### 2.1 Bioethics committee

The study was approved by the Bioethics Committee, resolution number: RNN/287/19/KE (13th June, 2019).

### 2.2 Group characteristics

Three groups of participants were established. The first comprised outpatients and inpatients diagnosed with severe asthma according to GINA 2023 Guidelines, who were recruited from the Department of Internal Medicine, Asthma, and Allergy, N. Barlicki University Clinical Hospital No. 1 in Lodz, Poland. The second group consisted of patients with COPD, classified grade GOLD 2 or 3 as B or E categories in GOLD 2024 Standards (37); they were recruited from the Specialist Outpatient Clinic of Pulmonary Diseases and Allergology or received treatment from the Department of Pulmonology and Allergology at the same hospital. The third group functioned as a control, composed of healthy volunteer subjects chosen from the general population. These individuals neither had, nor were in the process of being diagnosed with, any chronic lung diseases or neurodegenerative disorders. Clear and stringent inclusion and exclusion criteria were set for all participants.

The size of the groups was limited by financial constraints. The project was intended to serve as a pilot study to generate initial findings. No power calculation was conducted prior to the study.

### 2.3 Eligibility criteria

The following inclusion criteria were applied: over 18 years old, possess a diagnosis of either asthma (for Group 1) or COPD (for Group 2) confirmed based on GINA2023 (38) or GOLD 2023 (39) Guidelines established by a specialist, provide informed consent, and have both a blood sample available for molecular research and spirometry results on hand. In addition, the following exclusion criteria were applied: age under 18 years, no informed consent, could not be classified into one of the established groups, presence of severe obstructive airway diseases other than asthma or COPD, presence of a past medical history of neurodegenerative disease such as Alzheimer’s disease, Parkinson’s disease or dementia.

The volunteers for the control group had to meet all following eligibility criteria: age over 18 years old, signed informed consent and agreement to provide a blood sample for molecular analysis and undergo spirometry. The following exclusion criteria were also applied: presence of chronic obstructive or inflammatory lung diseases, diagnosis of neurodegenerative diseases, such as Alzheimer’s disease, Parkinson’s disease, dementia, multiple sclerosis, amyotrophic lateral sclerosis, mild cognitive impairment or any other degenerative cerebral disorder.

### 2.4 Examination procedure

A non-fasting blood sample was taken, and spirometry was performed as per ERS (40) technical standards. Additionally, the patient underwent a series of psychometric tests and fulfilled numerous questionnaires. In total, each participant completed sixteen questionnaires. The first part comprised eleven self-reported questions, scales and scores: 1) demographic data; several asthma-specific questionnaires recommended by the Global Strategy for Asthma Management and Prevention: 2) the Asthma Control Test TM (ACT TM) (39, 40) and 3) the Asthma Control Questionnaire (ACQ) (41, 42) and 4) the standardized Asthma Quality of Life Questionnaire (AQLQ(S)) (43).

The COPD patients completed several specific tools in line with Global Initiative for Chronic Lung Disease (GOLD) guidelines: 5) the COPD Assessment Test (CAT) (44, 45), 6) the modified Medical Research Council dyspnea scale (mMRC) (46), and 7) the St. George’s Respiratory Questionnaire specifically tailored for COPD patients (SGRQ-C) (47, 48).

Another three tests were used to describe the quality of life: 8) the 36-item Short Form Health Survey (SF-36) (49, 50), 9) The Index of Independence in Activities of Daily Living (ADL) (51, 52), and 10) The Lawton Instrumental Activities of Daily Living (IADL) (53).

The SF-36 results can be categorized into eight domains, and there are various methodologies for interpreting them. One renowned method is based on Taft’s Hypothesis (41), which calculates the result according to a *Physical Component Summary* (PCS) and a *Mental Component Summary* (MCS); in this case, scores above the expected range indicate not an improvement in health, but a decline in another health aspect (41). As this interpretation can be confusing, the present study adopted a different interpretation described in the *Methodology of quality of life assessment* (42) used in pharmacological care: this method organizes the eight domains into two components, using the median of each as a summary for physical and mental summaries.

The last self-reported test in the first part, 11) the GDS, counts to the indirect evaluation of cognitive functions.

The second part consist of observer-rated scales performed by the medical staff: 12) the Mini-Mental State Examination (MMSE) (43), which evaluates the cognitive aspects of mental functions (44), including mental tracking, expressive and receptive language, visual construction, immediate and delayed free verbal recall, calculation, and temporal and spatial orientation (45); 13) the Abbreviated Mental Test Score (AMTS) which assesses potential cognitive impairment (46), covering areas such as temporal and spatial orientation, visual memory, and semantic knowledge (45); 14) the Hachinski Ischaemic Score (HIS), which differentiates between senile or vascular origins of cognitive dysfunction (47–49); 15) the Clock Drawing Test (CDT), which is a cognitive screening tool used for the early detection, prediction, or monitoring of cognitive impairment (50, 51).

To evaluate deterioration in mental state, which can be both an indicator of cognitive decline and a hallmark of neurodegeneration (52–54), the following scales were implemented: 11) the 15-item Geriatric Depression Scale (GDS) (mentioned above), a self-rated tool that effectively screens for depression (55–57) and 16) the Hamilton Depression Rating Scale (HAM-D), which an observer-rated measure of depression (58, 59).

The testing procedure, comprising spirometry, blood sampling, and the sixteen tests, was conducted twice: first upon admission to the project and then again after a period of more than 12 months from the initial examination.

### 2.5 Biochemical methods

Venous blood samples from each patient were collected into tubes containing ethylenediaminetetraacetic acid (EDTA) and stored at -80°C.

#### 2.5.1 RNA extraction

Full blood was chosen as the most easily-available source of nucleated cells, i.e. white blood cells. Each full blood sample was pipetted into a separate 15 ml Falcon tube, at 2 ml of sample per tube, and 10 ml of RBCL cell lysis medium (A&A Biotechnology RBCL medium, REF 213-250, LOT 280222, Poland) were added to each tube. The tube contents were mixed and incubated on ice for 15 min. Tubes were centrifuged for 10 min at 3000 rpm and the supernatants discarded.

The remainder of the RNA extraction protocol was carried out with use of the A&A Biotechnology Total RNA Mini (REF 031-100, A&A Biotechnology, Poland) kit. Briefly, 800 µl of phenosol (a solution containing chaotropic salts and phenol) was added to each tube and cells lysed by repetitive pipetting. The tube contents were then transferred to sterile 1.5 ml Eppendorf tubes and incubated for 5 min at 50°C. Following this, 200 µl of chloroform was added, the samples were mixed gently, incubated for 3 min at RT, and then centrifuged for 10 min at 12 000 RPM. The supernatant was transferred to a new sterile 1.5 ml tube, and 250 µl of isopropanol was added to each tube.

The samples were then transferred to minicolumns placed in 2 ml tubes included in the RNA extraction kit, and centrifuged for 1 min at 12 000 RPM. The minicolumns were transferred to new 2 ml tubes and 700 µl of A1 wash solution were added to each minicolumn.

The samples were centrifuged for 1 min at 12 000 RPM. The filtrate was discarded from the tubes, the minicolumns were placed in the same tubes, 700 µl of A1 wash solution were added. The samples were centrifuged for 1 min at 12 000 RPM. The filtrate was discarded from the tubes, and the minicolumns were placed in the same tubes. Following this, 200 µl of A1 wash solution was added to each minicolumn. The samples were centrifuged for 2 min at 12 000 RPM.

The minicolumns were transferred to new sterile 1.5 ml elution tubes and 100 µl of sterile water was added directly onto the minicolumn silicon matrices. The samples were incubated for 3 min at room temperature (RT), centrifuged for 1 min at 12 000 RPM and the extracted RNA samples were stored at - 80°C until use.

#### 2.5.2 RNA concentration measurement

RNA concentrations were measured with the use of a Picodrop Spectrophotometer (60), which can detect RNA concentrations of 2 – 3000 μg/mL in 0.5-2.0 μL of solution without dilution (60).

#### 2.5.3 Reverse transcription

Reverse transcription was performed using the ImProm-II Reverse Transcription System (REF A3800, LOT 0000437833, Promega, USA).

The RNA samples were thawed on ice, diluted with nuclease-free water to final concentrations of 100 ng/µl, and 10µl of each sample was combined with 1.2 µl of Random Hex Primers in thin-walled PCR tubes. The tubes were placed into a preheated 70°C heat block for 5 minutes and immediately chilled on ice for another 5 minutes. The tubes were then centrifuged for 10 seconds and kept on ice until the later stages.

The 7.8 µl aliquots of the reverse transcription reaction mix (ImProm-II™ 5X Reaction Buffer 4.0 µl; dNTP Mix 1 µl; MgCl2 2.4 µl; ImProm-II™ Reverse Transcriptase 0.4 µl) were added to each PCR tube containing RNA samples and Random Hex primers (final volume 19 µl).

Annealing: The tubes were placed in a heat block at 25°C for 5 minutes.

Extension: The tubes were moved to a heat block at 42°C for one hour.

Reverse transcriptase inactivation: The tubes were moved to a heat block at 70°C for 15 minutes.

The resulting cDNA samples were stored at -20°C until subsequent stages of the assay.

#### 2.5.4 qPCR

The qPCR assay used the following reagents (all from Applied Biosystem, USA): Applied Biosystems TaqMan Universal PCR Master Mix REF 4304437 LOT 2208187; CREB-1 probe Hs00231713_m1 CREB1 LOT 1883523; PRKACB probe Hs01086757_m1 PRKACB LOT 1963364; Applied Biosystems Euk 18S rRNA (20X) REF 4333760F LOT 2003209. 

The cDNA samples were thawed and kept on ice.

The reaction mixes (Master Mix 7.5 µl; Nuclease-free water 6.25 µl; Experimental or 18S probe 0.75 µl.) were aliquoted into PCR tubes, 14.5 µl per tube.

Each experimental probe and the 18S rRNA (61) were assayed in duplicate reactions.

To each PCR tube was added 0.5 µl of patient cDNA. The PCR tubes were centrifuged for 10 seconds and placed in a Bio-Rad CFX96 Touch Real-Time PCR Detection System.

The following qPCR protocol was used: 50°C for 2 min, 95°C for 10 min, followed by 44 cycles comprising 95°C for 15 s, 60°C for 1 min and plate read (45 PCR cycles total).

After completing all the steps, the threshold cycle (CT) values for each sample were calculated using Mx-Pro software. For instances where the difference between the two technical CT replicates (for PKA, CREB, and the housekeeping reference gene, 18S) was ≤ 1 (61), an average was computed. The CT for each gene was then determined using the formula: ΔCT = C*_T, GENE_*– C*_T, 18S_*. If the difference between the values exceeded 1, the RT-PCR was performed again, and the same calculation was also repeated. The final ΔC*_T_* was derived as the mean of the initial examination and the repeated RT-PCR value. When 45 cycles were insufficient to detect the examined gene, the value was computed as the difference between the number of completed cycles and the C*_T, 18S_*. This method was employed in only 15 measurements. The acquired data were then entered into the database and subjected to statistical analysis.

### 2.6 Statistical analysis

Categorical variables were described as numbers and frequencies. They were compared between groups with the use of Pearson’s chi-square test or logistic regression. Ordinal variables were described as medians with 1^st^ and 3^rd^ quartiles and compared between groups with the Mann-Whitney *U*-test. Continuous variables were described as means and standard deviations, and were compared between groups with the Student’s *t*-test or one-way analysis of variance.

Cognitive and depression parameters were compared between patient groups as raw values and as values adjusted for potential confounding factors, *viz.* time between the 1^st^ and 2^nd^ examination, age, education level, residence, mental exercises (reading books, crosswords, Sudoku), underwent aesthesia, smoking pack years, BMI ≥25 kg/m^2^, therapy with systemic steroids. These covariates were included as linearly linked to the modeled parameters.

The analysis between two time-points was performed using the paired t-test. Linear regression was used to test for correlation between two continuous variables. P-values below 0.05 were considered statistically significant. The analyses were performed with STATISTICA 13.3 (StatSoft, Tulsa, OK, USA).

## 3 Results

### 3.1 Baseline characteristics

The study involved a total of 78 participants, divided into three distinct groups.

The asthma group comprised 11 women (42%) and 15 men (58%), mean age 55 years, while the COPD group consisted of 13 women (48%) and 14 men (52%), mean age 66 years. The age difference is consistent with the understanding that COPD typically manifests later in life compared to asthma (62, 63). Given potential confounding factors (64), such as age, all results were age-adjusted. The patients in both groups were administered medication according to the prevailing GINA and GOLD guidelines. This medication is summarized in **Supplementary Materials**, **Table 1**.

**Table 1:** Mean results in both groups at the first time point.

			Asthma *vs* CG		Asthma *vs* COPD		COPD *vs* CG	
Studied variable			raw	adjusted	raw	adjusted	raw	adjusted
CF	MMSE (higher better)	1^st^	**26.0 *vs* 27.6,** **p=0.014**	26.4 *vs* 27.2, p=0.16	26.0 *vs* 25.7, p=0.67	25.7 *vs* 25.9, p=0.87	**25.7 *vs* 27.6,** **p=0.006**	26.1 *vs* 27.1, p=0.31
	AMTS (higher better)	1^st^	**9.15 *vs* 9.64,** **p=0.024**	9.20 *vs* 9.60, p=0.059	9.15 *vs* 9.07, p=0.76	9.08 *vs* 9.23, p=0.62	**9.07 *vs* 9.64,** **p=0.016**	9.17 *vs* 9.63, p=0.15
	HIS (higher worse)	1^st^	1.46 *vs* 0.60, p=0.079	1.28 *vs* 0.84, p=0.40	1.46 *vs* 1.70, p=0.70	1.21 *vs* 2.07, p=0.33	1.70 *vs* 0.60, p=0.055	1.95 *vs* 0.41, p=0.10
	CDT	1^st^	**OR: 0.14 95%CI: 0.03-0.75,** **p=0.023**	OR: 0.04 95%CI: 0.001-1.01, p=0.051	OR: 0.85 95%CI: 0.27-2.70, p=0.77	OR: 0.19 95%CI: 0.01-2.72, p=0.21	**OR: 0.16 95%CI: 0.03-0.90,** **p=0.037**	OR: 0.17 95%CI: 0.01-4.71, p=0.29
PA	GDS (higher worse)	1^st^	**3.72 *vs* 1.48,** **p=0.014**	**3.51 *vs* 1.59,** **p=0.049**	3.72 *vs* 3.63, p=0.92	4.05 *vs* 3.11, p=0.44	**3.63 *vs* 1.48,** **p=0.001**	2.81 *vs* 2.19, p=0.52
	HAM-D (higher worse)	1^st^	**6.50 *vs* 3.04,** **p=0.010**	6.35 *vs* 3.33, p=0.060	6.50 *vs* 6.03, p=0.76	6.19 *vs* 6.63, p=0.84	**6.03 *vs* 3.04,** **p=0.013**	6.31 *vs* 2.92, p=0.088

Bold – statistically-significant values. The corresponding values for the second time point are presented in **Table 3** of the **Supplementary Material**.

The control group was composed of 14 women (56%) and 11 men (44%) with a mean age of approximately 53 years. Most of the individuals in this group were overweight or obese (56%). No other of reported diagnoses revealed statistically significant.

The participants were further evaluated through additional questionnaires and examinations. The most interesting results are presented in **Supplementary materials**, **Table 1**. The spirometry results confirmed the previously established diagnoses among the participants and the grouping arrangement. For the control group, the spirometry primarily confirmed the absence of any lung disease.

Of the original 78 participants, 66 were examined at the second time point. Of the 12 remainders, three, one from each group, passed away, seven withdrew (8.9% overall dropout rate) and two were lost to follow-up.

### 3.2 Cognitive functions evaluation

**Figure 2:**
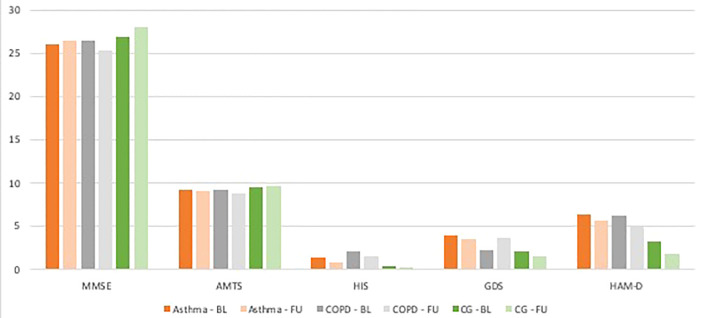
Mean adjusted results for each test within every group at the baseline and follow up. BL - baseline, FU - follow up.

In the raw analyses, the asthmatic and COPD patients presented poorer cognitive function than the control group, as revealed by MMSE, AMTS and CDT, and a higher prevalence of depressive symptoms according to the GDS and HAM-D. However, after adjusting for potential confounders, only borderline differences were found between asthmatic patients and controls with regard to depressive symptoms (GDS p=0.049), suggesting that asthma itself might predispose to depression (**Table 1**; **Figure 2**).

No notable differences were found between the asthma and COPD groups in terms of cognitive function tests or psychiatric assessments: no significant differences in raw and adjusted MMSE, AMTS, HIS, CTD, GDS, and HAM-D scored were observed between the two groups.

Before comparing the two time points, a test-retest analysis was conducted. The correlation of values between the two time points was calculated for MMSE (r=0.66), AMTS (r=0.56), HIS (r=0.57), CDT (r=0.73), GDS (r=0.77), and HAM-D (r=0.62). A significant result (p<0.05) was noted for the HAM-D results (p=0.036). This value suggests an improvement in psychiatric state over the test period.

The results of the questionnaires were assessed both within the entire study population and within each group at the first measurement point.

### 3.3 Linkage investigation

Regarding the asthma-specific tests, the ACT TM exhibited a moderate significant correlation with MMSE (R=0.598), AMTS (R=0.349), HIS (R=-0.405), GDS (R=-0.470), and HAM-D (R=- 0.380) in the study population. Within the asthma group, the only significant correlations of ACT TM were observed with direct assessment of cognitive functions (MMSE R=0.705, AMTS R=0.460, HIS R=-0.643). The ACQ correlated negatively with MMSE (R=-0.630) and AMTS (R=-0.362) in the general population, but positively with HIS (R=0.417), GDS (R=0.425), and HAM-D (R=0.369); however, in the asthma group, the ACQ only displayed a significant correlation with neurocognitive assessment (R=-0.612, R=-0.498, R=0.622 for MMSE, AMTS and HIS, respectively). Lastly, the AQLQ(S) was found to correlate with all questionnaires in the overall study population; within the asthma group, it correlated with HIS (R=-0.467), GDS (R=-0.511) and HAM-D (R=-0.489).

The COPD-specific tests, namely CAT, mMRC and SGRQ-C, were found to correlate with MMSE, AMTS, GDS, and HAM-D in the broader study population. In the COPD subgroup, the only significant relationships were found between CAT and MMSE (R=-0.542), HIS (R=0.433) or HAM-D (R=0.453) (**Table 2**).

**Table 2:** The correlations at the first time point.

	β	ACT TM	ACQ	AQLQ(S)	CAT	mMRC	SGRQ-C	FEV1[L]	FVC Ex [L]	PEF [L]	FEV/VC max
all participants*	MMSE	0.598	-0.630	0,520	-0.622	-0.472	-0.592	0.457	0.376	0.408	0.459
	AMTS	0.349	-0.362	0,327	NS	NS	-0.328	0.258	NS	NS	NS
	HIS	-0.405	0.417	-0.392	0.342	0.317	0.413	NS	NS	-0.250	-0.249
	GDS	-0.470	0.425	-0.472	0.432	0.574	0.491	-0.314	-0.277	NS	-0.247
	HAM-D	-0.380	0.369	-0.429	0.337	0.345	0.405	NS	-0.284	NS	NS
Asthma	MMSE	0.705	-0.612	NS	–	–	–	NS	NS	NS	0.553
	AMTS	0.460	-0.498	NS	–	–	–	NS	NS	NS	NS
	HIS	-0.643	0.622	-0.467	–	–	–	NS	NS	NS	-0.604
	GDS	NS	NS	-0.511	–	–	–	NS	NS	NS	NS
	HAM-D	NS	NS	-0.409	–	–	–	NS	NS	NS	NS
COPD	MMSE	–	–	–	-0.542	NS	-0.424	NS	NS	NS	NS
	AMTS	–	–	–	NS	NS	NS	NS	NS	NS	NS
	HIS	–	–	–	0.433	NS	NS	NS	NS	NS	NS
	GDS	–	–	–	NS	NS	NS	NS	NS	NS	NS
	HAM-D	–	–	–	0.453	NS	NS	NS	NS	NS	NS

NS, no statistical significance, p>0.05.

* Asthma + COPD + CG. The corresponding values for the second time point are presented in **Table 4** of the **Supplementary Material**.

MMSE, Mini-Mental State Examination; AMT, Abbreviated Mental Test; GDS, Geriatric Depression Scale; HAM-D, Hamilton Depression Rating Scale; COPD, chronic obstructive pulmonary disease; ACT TM, Asthma Control Test; ACQ, Asthma Control Test; AQLQ(S), The standardized Asthma Quality of Life Questionnaire; CAT, COPD Assessment Test; mMRC, modified Medical Research Council dyspnea scale; SGRQ, The St. George Respiratory Questionnaire dedicated COPD patients; FEV 1, forced expiratory volume in the first second; FVC Ex, forced vital capacity; PEF, peak expiratory flow; FEV/VC max, Tiffeneau-Pinelli index.

Furthermore, a baseline only 17 participants were able to accurately draw the clock in the asthma group, and only 13 patients in the COPD group. In contrast, 25 individuals in the control group executed the task correctly. Both comparisons with controls were significant (asthma vs. CG p=0.0055, COPD vs. CG p=0.0006).

Functional lung examination parameters, such as forced expiratory volume in the first second (FEV1), forced vital capacity (FVC Ex), peak expiratory flow (PEF), and the Tiffeneau-Pinelli index (FEV/VC max), correlated with all neuropsychiatric tests (MMSE, AMTS, GDS, and HAM-D) in the broader study population. However, the only notable relationship within the asthma group was between FEV/VC max and MMSE (R=0.553) or HIS (R=-0.604), and no correlation was found in the COPD group.

### 3.4 mRNA expression correlations

No correlation was found between the first and second time points for CREB mRNA expression at **Supplementary Materials**, **Table 2**; however, a 2.3-fold increase in CREB mRNA expression was observed between the two time points in study population (p=0.014). This change did not differ significantly between groups. When data from both time points were combined, a significant association was found between CREB and MMSE (β=0.166, p=0.048) (**Table 3**). In a model adjusted for covariates, a borderline interaction was noted between diagnosis and CREB in predicting MMSE (p=0.055). CREB mRNA level was significantly associated with MMSE in the COPD group (β=0.273, p=0.034), but this association was not significant in other groups. Among the other tests, CREB mRNA expression was significantly associated with AMTS, but only in the COPD group (p=0.018, β=0.333), and this association remained significant in the covariate-adjusted model (p=0.024, β=0.327).

**Table 3:** The relationships between pooled results from cognitive function tests and psychiatric assessment scores from both time-points and mRNA expression of the CREB gene.

Δ C*_T CREB_*		all participants*		Asthma		COPD		CG	
		β	p	β	p	β	p	β	p
MMSE	raw	**0.166**	**0.048**	-0.012	0.939	**0.337**	**0.017**	0.091	0.549
	adjusted	0.106	0.121	-0.029	0.819	**0.273**	**0.034**	0.086	0.460
AMTS	raw	0.105	0.210	-0.217	0.143	**0.333**	**0.018**	0.039	0.795
	adjusted	0.079	0.325	-0.212	0.169	**0.327**	**0.024**	0.001	0.993
HIS	raw	0.008	0.926	0.034	0.821	0.080	0.581	-0.090	0.551
	adjusted	-0.006	0.941	0.113	0.563	0.008	0.959	-0.259	0.173
GDS	raw	-0.005	0.949	0.112	0.460	-0.138	0.338	0.067	0.658
	adjusted	0.056	0.499	0.128	0.454	0.032	0.859	0.075	0.607
HAM-D	raw	-0.051	0.542	0.087	0.561	-0.185	0.198	0.008	0.959
	adjusted	-0.007	0.934	0.127	0.453	-0.095	0.622	-0.019	0.890

* Asthma + COPD + CG.

Bold – Statistically-significant values.

Cognitive functions: MMSE, Mini-Mental State Examination; AMT, Abbreviated Mental Test; HIS, Hachinski Ischaemic Score; Psychiatric assessment: GDS, Geriatric Depression Scale; HAM-D, Hamilton Depression Rating Scale; COPD, chronic obstructive pulmonary disease; CG, control group, Δ C_T_, delta cycle threshold values.

A logistic regression was conducted to identify whether the CDT results (**Table 4**), as an indicator of cognitive dysfunction, depended on the mRNA expression for both genes. All results obtained were statistically insignificant (p>0.05). Contrary to the previously described associations between CREB and MMSE or AMTS, no relationship was found between CREB and CDT in any group. In addition, no significant associations were found between CDT and PKA mRNA expression.

**Table 4:** The results of logistic regression of the associations between pooled CDT test results and ΔC*_T CREB_* and ΔC*_T PKA_* expression between research cohorts.

CDT	Δ C*_T CREB_*				Δ C*_T PKA_*			
	all	Asthma	COPD	CG	all	Asthma	COPD	CG
OR	0.976	1.046	0.887	0.445	0.946	0.866	1.246	0.969
-95%CL	0.831	0.844	0.671	0.136	0.820	0.707	0.911	0.517
+95%CL	1.146	1.295	1.171	1.459	1.092	1.061	1.704	1.821
p	0.767	0.675	0.384	0.169	0.445	0.155	0.157	0.922

CDT, Clock Drawing Test; COPD, chronic obstructive pulmonary disease; CG, control group; Δ C_T_, delta cycle threshold values.

Raw data.

PKA mRNA expression was compared between the two time points. A significant correlation was observed only in the COPD group (p=0.049, β=-0.4157). Intriguingly, no significant change in PKA mRNA expression was noted between these points, mirroring the findings for CREB expression. In addition, no differences in PKA mRNA expression were identified between groups. Additionally, PKA mRNA level was not linked to any cognitive function test or psychiatric assessment (**Table 5**). Even when pooling the results, no statistical significance emerged. Similarly, the covariate-adjusted model yielded no significant findings.

**Table 5:** Summary of correlations between combined cognitive test results and psychiatric evaluations in groups organized by ΔC_T PKA_ expression level in the studied cohorts.

Δ C*_T PKA_*		all participants*		Asthma		COPD		CG	
		β	p	β	p	β	p	β	p
MMSE	raw	-0.078	0.356	-0.111	0.456	-0.084	0.562	0.073	0.632
	adjusted	-0.066	0.319	-0.048	0.702	-0.153	0.194	0.012	0.917
AMTS	raw	-0.022	0.889	0.053	0.722	-0.041	0.779	-0.007	0.965
	adjusted	-0.014	0.859	0.121	0.436	-0.165	0.216	0.029	0.846
HIS	raw	-0.053	0.531	-0.153	0.304	-0.090	0.533	0.168	0.264
	adjusted	-0.062	0.472	-0.170	0.257	-0.043	0.786	0.191	0.195
GDS	raw	0.025	0.768	0.037	0.808	0.015	0.917	0.161	0.435
	adjusted	0.021	0.793	0.067	0.695	-0.014	0.929	-0.135	0.350
HAM-D	raw	0.066	0.435	0.058	0.696	0.009	0.951	0.043	0.776
	adjusted	0.068	0.428	0.194	0.246	-0.066	0.703	0.065	0.635

Bold – statistically-significant values.

* Asthma + COPD + CG.

Cognitive functions: MMSE, Mini-Mental State Examination; AMT, Abbreviated Mental Test; HIS, Hachinski Ischaemic Score; Psychiatric assessment: GDS, Geriatric Depression Scale; HAM-D , Hamilton Depression Rating Scale; COPD, chronic obstructive pulmonary disease; CG, control group, Δ C_T_, delta cycle threshold values.

Remarkably, a relationship between PKA and CREB mRNA expression was evident at both the initial time point (p=0.000, β=0.610) and the later one (p=0.001, β=0.411). At baseline, a significant association between PKA and CREB was observed in the COPD group (p=0.000, β=0.929); however, at the second time point, a significant association was noted for both the asthma group (p=0.016, β=0.506) and the COPD group (p=0.028, β=0.458).

## 4 Discussion

At first glance, chronic obstructive inflammatory pulmonary diseases appear to be a heterogeneous group with diversified endotypes. For example, asthma and COPD are characterized by different risk factors, and while inflammation may be the primary underlying mechanism in both diseases, the two differ with regard to the cells involved.

Several studies have examined whether inflammation might be a key factor influencing neurodegeneration in the hippocampus, and their findings suggest that better respiratory function in mid-life may be linked to a reduced risk of developing neurodegenerative diseases such as Alzheimer’s disease, Parkinson’s disease, mild cognitive impairment, frontotemporal dementia or lewy body dementia in later life (31). Indeed, other atopic conditions such as eczema and rhinitis have also been associated with a modest increase in the risk of neurodegenerative dementia which was not attributed to vascular disease or genetic factors (65); however, the frequency of asthma was not found to be higher among a cohort of dementia patients compared to controls (66).

A national cohort study also indicated a relationship between poor pulmonary function and cognitive impairment (31), which was also supported by pathomorphological examinations. Also, chronic asthma has been shown to lead to damage in the hippocampal ultrastructure of mice (67). While a temporary state of hypoxia directly followed by reoxia has been found to increase activation of MAPK (68, 69), there is currently no data about the effect of long-term hypoxia accompanied by inflammation on brain tissue, nor on the molecular link between asthma or COPD, being typical chronic obstructive lung diseases, and cognitive dysfunction. As such, the present article is one of the first to look for the association between proteins and these conditions in the neurodegenerative processes; it is also the first to prospectively monitor patients with chronic inflammatory diseases and consider the results in the light of data from sensitive tests for the diagnosis of dementia.

The demographic data collected at the study onset revealed initial distinctions among the groups. Notably, those with asthma tended to have lower educational attainment compared to controls, as well as lower quality of life in several domains based on the SF-36. Also, the asthma patients were more frequently employed and less prone to smoking than the COPD patients.

Further disparities emerged when cognitive functions were assessed by questionnaires. For example, a notable difference was found between the asthma and control groups concerning the deterioration of cerebrovascular parameters, as determined by AMTS and CDT; this suggests that asthma significantly hampers higher cognitive functions. Even after adjusting for the previously mentioned covariates, the results remained markedly lower for the asthma group compared to the controls. The presence of inferior cognitive function in asthma patients compared to controls raises the possibility that this defect may be associated with chronic obstructive lung disease. Indeed, no significant distinction in cognitive ability was noted between the asthma and COPD groups, suggesting that the nature of inflammation in chronic obstructive pulmonary disease does not play a pivotal role in neurodegenerative progression. This insight is clinically significant.

Additionally, no clear differences in cognitive functions, assessed using the MMSE tool, were found between the asthma and CG groups. Furthermore, while asthma and COPD progress along different inflammatory pathways, neither seems to influence cognitive dysfunction or neurodegeneration; in addition, neither condition appears to be associated with cognitive disturbances or increased risk of neurodegeneration. To elucidate these connections, more comprehensive research is warranted.

Among the asthma group, diminished cognitive function seems to manifest primarily as depression. The psychiatric status of the studied patients varied over the observation period, with no clear improvement in cognition identified based on the HAM-D outcomes. Interestingly, no association of mental status with any specific disease could be discerned from these results. Perplexingly, no notable shift in cognitive function was observed between the first and second time points for either asthma or COPD patients. One possible explanation for this static observation could be the relatively short duration of the study, suggesting that a more extended observation period might provide clearer insights.

Two molecular factors associated with the development of Parkinson’s disease (70), were selected for study as potential indicators of neurodegeneration in chronic obstructive inflammatory pulmonary disease: PKA and CREB. These were identified by a detailed analysis of the GLP-1 agonist mechanism (16). PKA mRNA levels did not exhibit any correlation with cognitive dysfunction. In contrast, CREB mRNA emerged as a potential marker of cognitive decline, specifically in COPD patients. Limitations of this study include the consideration of only two signaling factors in the GLP-1R signaling pathway. Analyzing all possible biomarkers exceeds the scope of this work and will be included in future prospective studies. Another limitation to note is the pilot nature of the study. Despite estimating the minimum sample size using an ANOVA model with a medium effect size (Cohen’s f = 0.25), the feasible number of subjects was recruited. This could lead to underpowered findings and increases the risk of type II errors. Consequently, detecting more subtle effects and differences might be hinder. Additionally, due to the small size of the groups, age-matched controls could not be provided. To minimize the impact of age discrepancies, all analyses were age-adjusted.

There is a need for more extensive and prolonged research to identify more potential markers of neurodegeneration among those associated with inflammation, a more extensive and prolonged study might be necessary.

## Data availability statement

The original contributions presented in the study are included in the article/**Supplementary Material**. Further inquiries can be directed to the corresponding author.

## Ethics statement

The studies involving humans were approved by Bioethics committee of Medical University of Lodz. The studies were conducted in accordance with the local legislation and institutional requirements. The participants provided their written informed consent to participate in this study.

## Author contributions

MF: Conceptualization, Investigation, Methodology, Project administration, Resources, Visualization, Writing – original draft, Writing – review & editing. AW: Investigation, Resources, Writing – review & editing. JP: Investigation, Resources, Writing – review & editing. JD: Project administration, Resources, Writing – review & editing. SM: Project administration, Resources, Writing – review & editing. MK: Data curation, Formal analysis, Validation, Writing – review & editing. PK: Funding acquisition, Project administration, Writing – review & editing. MP: Conceptualization, Methodology, Project administration, Supervision, Validation, Writing – review & editing.

## Glossary

**Table:**

ΔCT	delta cycle threshold values
18S rRNA	Ribosomal ribonucleic acid of 18S subunit
ACE	angiotensin-converting enzyme inhibitors
ACQ	Asthma Control Questionnaire
ACT TM	Asthma Control Test TM
ADL	The Index of Independence in Activities of Daily Living
AMT	Abbreviated Mental Test
AQLQ(S)	The standardized Asthma Quality of Life Questionnaire
ASM	airways smooth muscles
BMI	Body Mass Index
cAMP	cyclic adenosine monophosphate
CAT	COPD Assessment Test
cDNA	complementary DNA
CDT	Clock Drawing Test
CF	Cognitive functions
CG	control group
COPD	chronic obstructive pulmonary disease
CREB	cAMP response element binding protein
CREB-1	the gene responsible for transcription of all cAMP-responsive element-binding protein isoforms
dNTP	deoxynucleotide triphosphate
EDTA	ethylenediaminetetraacetic acid
Ex-4	exendin-4
f	female
GDS	Geriatric Depression Scale
GLP-1R	glucagon-like peptide-1 receptor
HAM-D	Hamilton Depression Rating Scale
HIS	Hachinski Ischaemic Score
IADL	The Lawton Instrumental Activities of Daily Living
ICS	inhaled corticosteroids
ICS	inhaled corticosteroids
IgE	immunoglobulin E
IL-1β	interleukin-1β
IL-10	interleukin-10
IL-5	interleukin-5
IL-6	interleukin-6
K	thousand
LABA	Acting Beta Agonists
LTRA	leukotriene receptor antagonists
m	masculine
MAPK	p38 mitogen activated protein kinase
MCS	Mental Component Summary
mMRC	modified Medical Research Council dyspnea scale
MMSE	Mini-Mental State Examination
NF-κB	nuclear factor-κB
NS	no statistical significance, p>0.05
PA	Psychiatric assessment
PCR	Polymerase Chain Reaction
PCS	Physical Component Summary
PKA	protein kinase A
PRKACB	cAMP-dependent protein kinase catalytic subunit beta
qPCR	quantitative polymerase chain reaction
RANTES (or CCL5)	chemokine regulated upon activation, normal T cell expressed and secreted
RNA	Ribonucleic acid
RPM	rotations per minutes
RT	room temperature
RT-PCR	Real-Time PCR
SABA+LAMA	short-acting beta-agonists combined with long-acting muscarine antagonists
SF-36	the 36-item Short Form Health Survey
SGRQ-C	The St. George Respiratory Questionnaire dedicated COPD patients
Th2	CD4+ T-helper 2 Lymphocytes
