# Supplementary material for: Potential association between obstructive lung diseases and cognitive decline
Source: Front Immunol. 2024 Jul 22;15:1363373. doi: 10.3389/fimmu.2024.1363373 (PMC11298337; doi:10.3389/fimmu.2024.1363373)
Supplement: Supplementary file 1 [file Table_1.docx]

Supplementary Material

Potential linkage between obstructive lung diseases and cognitive decline.

Magdalena Figat^1^, Aleksandra Wiśniewska^2^, Jacek Plichta^1^, Joanna Dymanowska-Miłkowska^3^, Sebastian Majewski^3^, Michał S. Karbownik^4^, Piotr Kuna^1^, Michał G. Panek^1^

*** Correspondence:** Magdalena Figat, magdalena.figat@gmail.com

# Supplementary Figures and Tables

Table 1. Demographic description of both groups.

| Parameters | | Mean and SD or % | | | | | | | | p | | | | |
| --- | --- | --- | --- | --- | --- | --- | --- | --- | --- | --- | --- | --- | --- | --- |
|  |  | Asthma | | | COPD | | | CG | | Asthma-COPD | Asthma-CG | | COPD-CG | |
| N | | 26 | | | 27 | | | 25 | |  |  |  |  |  |
| Sex: f vs m | | 11, 42%  *vs* 15, 58% | | | 13, 48%  *vs* 14, 52% | | | 14, 56%  *vs* 11, 44% | | 0.67 | 0.33 | | 0.57 | |
| Age [years] | | 55 | | 16.45 | 66.19 | | 7.61 | 53.2 | 14.47 | **0.002** | 0.68 | | **<0.001** | |
| BMI [kg/m^2^] | | 27.68 | | 5.35 | 26.01 | | 3.86 | 27.01 | 3.19 | 0.20 | 0.59 | | 0.32 | |
| Residence: | | | | | | | | | | 0.11 | 0.18 | | **0.007** | |
| - village | | 15% | | | 4% | | | 32% | |  |  | |  | |
| - town to 100 thousand citizens | | 31% | | | 11% | | | 28% | |  |  |  |  |  |
| - between 100 and 500 thousand citizens | | 4% | | | 7% | | | 8% | |  |  |  |  |  |
| - over 0,5 mln citizens | | 50% | | | 78% | | | 32% | |  |  |  |  |  |
| Household size | | 2 | | | 1 or 2 | | | 2 | | 0.31 | 0.49 | | **0.072** | |
| Educational level: | | | | | | | | | | 0.47 | **0.047** | | **0.009** | |
| - elementary school | | 4% | | | 11% | | | 4% | |  |  | |  | |
| - vocational school | | 27% | | | 30% | | | 4% | |  |  |  |  |  |
| - technical vocational school | | 42% | | | 33% | | | 36% | |  |  |  |  |  |
| - high school | | 19% | | | 26% | | | 52% | |  |  |  |  |  |
| - higher education | | 8% | | | 0 | | | 4% | |  |  |  |  |  |
| Salary: | | | | | | | | | | 0.44 | 0.17 | | **0.036** | |
| <2 K PLN | | 29% | | | 39% | | | 13% | |  |  | |  | |
| 2 – 3 K PLN | | 37% | | | 35% | | | 39% | |  |  |  |  |  |
| 3 – 4 K PLN | | 13% | | | 17% | | | 13% | |  |  |  |  |  |
| 4 – 5 K PLN | | 13% | | | 4% | | | 17% | |  |  |  |  |  |
| 5 – 6 K PLN | | 4% | | | 4% | | | 4% | |  |  |  |  |  |
| > 6 K PLN | | 4% | | | 0 | | | 13% | |  |  |  |  |  |
| Professional activity | | 54% | | | 23% | | | 64% | | **0.02** | 0.46 | | **0.003** | |
| White-collar job | | 46% | | | 42% | | | 64% | | 0.78 | 0.20 | | 0.12 | |
| Cigarette consumption currently | | 2, 8%  py/person=12,68  Ex-Smokers:  8, 31%  py/person=24,11 | | | 13, 48%  py/person=45,55    Ex-Smokers:  12, 44% py/person=56,64 | | | 2, 8%  py/person=30,63  Ex-Smokers:  9, 36%  py/person=9,91 | | **0.001** | 1 | | **0.001** | |
| Social alcohol consumption | | 63% | | | 88% | | | 87% | | **0.04** | 0.07 | | 0.87 | |
| Mental exercises | | 81% | | | 85% | | | 88% | | 0.73 | 0.70 | | 1.00 | |
|  | | | | | | | | | | | | | | |
| Past Medical History | | | | | | | | | | | | | | |
| - Type 2 Diabestes | | 15% | | | 30% | | | 4% | | 0.22 | | 0.17 | | **0.01** |
| - Hypertension | | 38% | | | 70% | | | 20% | | **0.02** | | 0.15 | | **<0.001** |
| - Heart failure | | 12% | | | 15% | | | 0 | | 0.72 | | 0.08 | | **0.045** |
| - Post-MI | | 8% | | | 7% | | | 4% | | 0.97 | | 0.58 | | 0.6 |
| - IHD | | 4% | | | 15% | | | 4% | | 0.17 | | 0.98 | | 0.19 |
| - Arrythmia | | 8% | | | 11% | | | 12% | | 0.67 | | 0.61 | | 0.92 |
| - Arthritis/RA | | 4% | | | 4% | | | 4% | | 0.98 | | 0.98 | | 0.96 |
| - Any allergy | | 62% | | | 44% | | | 28% | | 0.21 | | **0.02** | | 0.22 |
| - Underwent surgery | | 65% | | | 81% | | | 40% | | 0.319 | | 0.07 | | **0.002** |
|  | | | | | | | | | | | | | | |
| Medication | | | | | | | | | | | | | | |
|  | |  | | |  | | |  | | Asthma *vs* COPD | | | | |
| ICS+LABA | | 80% | | | 23% | | | 0 | | 0.00005 | | | | |
| ICS | | 48% | | | 8% | | | 0 | | 0.001 | | | | |
| antihistamines | | 52% | | | 4% | | | 0 | | 0.00012 | | | | |
| LTRA | | 28% | | | 4% | | | 0 | | 0.0024 | | | | |
| immunotherapies | | 42% | | | 0% | | | 0 | | 0.00019 | | | | |
| β-antagonists | | 16% | | | 58% | | | 0 | | 0.00208 | | | | |
| SABA+LAMA | | 0% | | | 27% | | | 0 | | 0.22 | | | | |
| vasodilators | | 0% | | | 23% | | | 0 | | 0.024 | | | | |
| ACE | | 4% | | | 31% | | | 0 | | 0.024 | | | | |
|  | | | | | | | | | | | | | | |
| Assessment of life quality | | | | | | | | | | | | | | |
| SF-36 | PCS | 51.83 | | 25.34 | 42.68 | | 21.71 | 75.61 | 14.31 | 0.16 | **<0.001** | | **<0.001** | |
|  | MCS | 62.08 | | 25.94 | 57.64 | | 20.54 | 75.92 | 15.26 | 0.49 | **0.025** | | **<0.001** | |
| ADL | | 6 | | - | 5.89 | | 0.58 | 6 | - | 0.33 | - | | 0.34 | |
| IADL | | 23.3 | | 1.35 | 22.59 | | 1.93 | 23.84 | 0.47 | 0.13 | 0.07 | | **0.002** | |
|  | | | | | | | | | | | | | | |
| Asthma Assessment – performed only in Asthma Group | | | | | | | | | | | | | | |
|  | | Mean | SD | |  | | | | | | | | | |
| ACT TM | | 16.17 | 6.26 | |  |  |  |  |  |  |  |  |  |  |
| ACQ | | 14.77 | 9.38 | |  |  |  |  |  |  |  |  |  |  |
| AQLQ(S) | | 4.71 | 1.44 | |  |  |  |  |  |  |  |  |  |  |
|  | | | | | | | | | | | | | | |
| COPD Assessment – performed only in COPD Group | | | | | | | | | | | | | | |
|  | |  | | | Mean | SD | |  | | | | | | |
| CAT | |  |  |  | 20.85 | 8.1 | |  |  |  |  |  |  |  |
| mMRC | |  |  |  | 1.85 | 1.17 | |  |  |  |  |  |  |  |
| SGRQ-C | |  |  |  | 55.11 | 20.61 | |  |  |  |  |  |  |  |

**Bold** – statistically-significant values.
Detailed data presented in the text. The author’s own analysis

COPD – chronic obstructive pulmonary disease, CG – control group, f – female, m – masculine, K – thousand, BMI – Body Mass Index, ICS – inhaled corticosteroids, LABA-Acting Beta Agonists, LTRA – leukotriene receptor antagonists, SABA+LAMA – short-acting beta-agonists combined with long-acting muscarine antagonists, ACE – angiotensin-converting enzyme inhibitors, SF-36 – the 36-item Short Form Health Survey, PCS - Physical Component Summary, MCS – Mental Component Summary, ADL - The Index of Independence in Activities of Daily Living, IADL – The Lawton Instrumental Activities of Daily Living, ACT TM – Asthma Control Test TM, ACQ – Asthma Control Questionnaire, AQLQ(S) – The standardized Asthma Quality of Life Questionnaire, CAT – COPD Assessment Test, mMRC – modified Medical Research Council dyspnea scale, SGRQ-C – The St. George Respiratory Questionnaire dedicated COPD patients, SD – Standard deviation.

Table 2. Average the 2(-$\Delta$CT) qPCR measurement of CREB and PKA expression at baseline and follow up in the three study groups with normalization by 18S rRNA.

|  | Asthma | | COPD | | CG | |
| --- | --- | --- | --- | --- | --- | --- |
|  | baseline | follow up | baseline | follow up | baseline | follow up |
| PKA | -13.94±1.21 | -13.70±4.90 | -13.92±2.04 | -12.24±2.37 | -13.20±1.87 | -12.87±2.82 |
| CREB | -13.62­±2.20 | -11.42±3.34 | -13.28±2.28 | -12.33±2.82 | -12.29±1.66 | -12.26±2.01 |

Table 3. Average results in both groups at the second time point.

|  | |  | Asthma *vs* CG | | Asthma *vs* COPD | | COPD *vs* CG | |
| --- | --- | --- | --- | --- | --- | --- | --- | --- |
| Studied variable | | | raw | adjusted | raw | adjusted | raw | adjusted |
| CF | MMSE (higher better) | 2^nd^ | 26.45 *vs* 28.00  p=0.08 | 27.34 *vs* 27.51  p=0.79 | 26.45 *vs* 25.34  p=0.30 | 26.29 *vs* 25.85  p=0.66 | **25.34 *vs* 28.00**  **p=0.002** | 26.14 *vs* 27.12  p=0.41 |
|  | AMTS (higher better) | 2^nd^ | **9.04 *vs* 9.71**  **p=0.005** | **9.11 *vs* 9.78**  **p=0.004** | 9.04 *vs* 8.73  p=0.40 | 9.06 *vs* 8.85  p=0.61 | **8.73 *vs* 9.71**  **p=0.006** | 8.91 *vs* 9.52  p=0.25 |
|  | HIS (higher worse) | 2^nd^ | **0.90 *vs* 0.28**  **p=0.02** | 0.82 *vs* 0.40  p=0.16 | 0.90 *vs* 1.52  p=0.13 | 1.05 *vs* 1.43  p=0.49 | **1.52 *vs* 0.28**  **p=0.002** | 1.47 *vs* 0.33  p=0.09 |
|  | CDT | 2^nd^ | 0-NaN* | **-** | OR: 0.59 95%CI: 0.15-2.35  p=0.44 | OR: 0.15 95%CI: 0.009-2.37  p=0.16 | 0-NaN* | - |
| PA | GDS (higher worse) | 2^nd^ | **3.54 *vs* 1.52**  **p=0.04** | 3.37 *vs* 1.71  p=0.13 | 3.54 *vs* 3.65  p=0.91 | 3.83 *vs* 3.41  p=0.75 | **3.65 *vs* 1.52**  **p=0.009** | 3.60 *vs* 1.57  p=0.12 |
|  | HAM-D (higher worse) | 2^nd^ | **5.63 *vs* 1.90**  **p=0.006** | **5.82 *vs* 1.98**  **p=0.01** | 5.63 *vs* 4.95  p=0.66 | 6.13 *vs* 4.74  p=0.46 | **4.95 *vs* 1.90**  **p=0.009** | 4.34 *vs* 2.57  p=0.37 |

**Bold** – statistically-significant values.
Detailed data presented in the text. The author’s own analysis.

CG – control group, COPD – chronic obstructive pulmonary disease, CF – cognitive functions: MMSE – Mini-Mental State Examination, AMT – Abbreviated Mental Test, HIS – Hachinski Ischaemic Score, CDT – Clock Drawing Test; PA – psychiatric assessment: GDS – Geriatric Depression Scale, HAM-D – Hamilton Depression Rating Scale, NaN – Not a number.

*All participants in CG performed CDT correctly.

Table 4. The found correlations at the follow up.

|  | β | ACT TM | ACQ | AQLQ(S) | CAT | mMRC | SGRQ-C | FEV1[L] | FVC Ex [L] | PEF [L] | FEV/VC max |
| --- | --- | --- | --- | --- | --- | --- | --- | --- | --- | --- | --- |
| all participants* | MMSE | 0.401 | -0.445 | 0.439 | -0.472 | -0.468 | -0.461 | 0.496 | 0.442 | 0.500 | 0.455 |
|  | AMTS | 0.294 | -0.337 | 0.299 | -0.350 | -0.344 | -0.332 | 0.402 | 0.379 | 0.397 | 0.364 |
|  | HIS | -0.331 | 0.310 | -0.444 | 0.411 | 0.471 | 0.487 | -0.358 | -0.341 | NS | -0.328 |
|  | GDS | -0.446 | 0.494 | -0.503 | 0.423 | 0.460 | 0.538 | -0.394 | -0.391 | -0.323 | -0.263 |
|  | HAM-D | -0.286 | 0.348 | -0.377 | 0.389 | 0.389 | 0.408 | -0.359 | -0.348 | -0.267 | NS |
| Asthma | MMSE | NS | -0.423 | NS | - | - | - | 0.452 | NS | 0.514 | 0.716 |
|  | AMTS | NS | NS | NS | - | - | - | NS | NS | 0.450 | NS |
|  | HIS | NS | NS | NS | - | - | - | NS | NS | NS | NS |
|  | GDS | -0.507 | 0.577 | -0.616 | - | - | - | -0.444 | NS | NS | NS |
|  | HAM-D | NS | NS | NS | - | - | - | NS | NS | NS | NS |
| COPD | MMSE | - | - | - | NS | NS | NS | NS | NS | NS | NS |
|  | AMTS | - | - | - | NS | NS | NS | NS | NS | NS | NS |
|  | HIS | - | - | - | 0.475 | 0.432 | 0.498 | NS | NS | NS | NS |
|  | GDS | - | - | - | 0.428 | NS | 0.420 | NS | NS | NS | NS |
|  | HAM-D | - | - | - | NS | NS | NS | NS | NS | NS | NS |

Detailed data presented in the text. The author’s own analysis. NS – no statistical significance, p>0.05

* Asthma + COPD + CG

MMSE – Mini-Mental State Examination, AMT – Abbreviated Mental Test, GDS – Geriatric Depression Scale, HAM-D – Hamilton Depression Rating Scale; COPD – chronic obstructive pulmonary disease, ACT TM – Asthma Control Test, ACQ – Asthma Control Test, AQLQ(S) – The standardized Asthma Quality of Life Questionnaire, CAT – COPD Assessment Test, mMRC – modified Medical Research Council dyspnea scale, SGRQ – The St. George Respiratory Questionnaire dedicated COPD patients, FEV 1 – forced expiratory volume in the first second, FVC Ex – forced vital capacity, PEF – peak expiratory flow, FEV/VC max – Tiffeneau-Pinelli index
